# Reactive, Proactive, Relational, and Slow Dissipation of Aggression in Children

**DOI:** 10.1016/j.jaacop.2023.12.007

**Published:** 2023-12-29

**Authors:** Daniel A. Waschbusch, Susan D. Mayes, James G. Waxmonsky, Dara E. Babinski, Raman Baweja

**Affiliations:** a Penn State College of Medicine, Hershey, Pennsylvania

**Keywords:** aggression subtypes, callous-unemotional traits, irritability, children, sex differences

## Abstract

**Objective:**

This study investigated the associations between aggression subtypes (reactive, proactive, relational, and slow dissipation of aggression), callous-unemotional traits (CU), irritability (IRR), and sex among children.

**Method:**

The sample included 508 children 5 to 12 years of age, rated by their mothers.

**Results:**

A 4-factor model of aggression subtypes (reactive, proactive, relational, slow dissipation) provided a good fit to the data. Correlations between aggression subtypes and demographic variables were generally nonsignificant, except for a unique association between relational aggression and sex, with girls exhibiting higher scores, and proactive aggression was associated with younger age. Both CU and IRR correlated positively with all types of aggression. Slow dissipation of aggression showed a unique association with IRR, whereas reactive aggression was uniquely associated with both CU and IRR. Regressions showed an interaction between CU, IRR, and sex for slow dissipation and relational aggression. Interactions showed that boys with high levels of both CU and IRR demonstrated slower dissipation of aggression, indicating a tendency to hold grudges, and that sex differences in relational aggression depended on the co-occurrence of CU and IRR.

**Conclusion:**

This study sheds light on the interplay among aggression subtypes, CU, IRR, and sex in children. Findings emphasize the need for psychiatrists and other mental health professionals to consider the multifaceted nature of aggressive behavior and the role of CU and IRR when assessing aggressive children or developing treatment plans for them. Further research is warranted to examine these relationships longitudinally and across diverse populations.

**Clinical guidance:**

• Recognize the different types of aggression: Look beyond angry outbursts and consider reactive, proactive, relational, and slow dissipation of aggression to target intervention.
• Emotional traits matter: Assess for callous-unemotional traits and irritability alongside aggression. High levels of both, especially in boys, may indicate greater difficulty managing anger and holding grudges.
• Gender differences exist: Girls may be more prone to relational aggression compared to boys.
• Proactive aggression might be more common in younger than in older children. Tailor interventions accordingly.

## Plain language summary

In this study of 508 children between age 5 to 12, maternal ratings were used to distinguish 4 different types of aggression in their children: reactive (like hitting back when teased), proactive (starting fights), relational (spreading rumors), and slow to cool down (holding grudges). Girls were found to be more relational aggressors, and proactive aggression was more common in younger children. Results also showed that children with certain emotional traits (callous and irritable) were more likely to show all types of aggression, and boys who were both callous and irritable held onto their anger. All 4 types of aggression were associated with externalizing disorders: attention-deficit/hyperactivity disorder, oppositional defiant disorder, and conduct disorder.

Childhood aggression presents a complex and multifaceted phenomenon that can have severe consequences for children and society^1^ and often prompts parents to seek mental health services.^2^ Understanding the different types of aggression and factors that influence their development and expression is crucial for developing effective prevention and intervention strategies.^3^^,^^4^ In this study, we examine 4 types of aggression in children 5 to 12 years of age: reactive, proactive, relational, and slow dissipation of aggression.

Reactive and proactive aggression are 2 significant subtypes of aggression that differ in their motives and goals.^5^ Reactive aggression refers to impulsive, angry responses elicited by perceived provocation, whereas proactive aggression refers to a premeditated and instrumental use of aggression to achieve specific goals. These subtypes are distinct but correlated, with an average correlation of 0.68 in 1 meta-analysis.^6^ Reactive aggression exhibits stronger correlations than proactive aggression with attention-deficit/hyperactivity disorder (ADHD), internalizing psychopathology (such as anxiety and depression), and peer rejection. In contrast, proactive aggression exhibits stronger correlations with conduct disorder and callous-unemotional traits.^6^^,^^7^

Relational aggression, also known as social aggression,^8^ refers to behaviors that harm others through social manipulation, such as spreading rumors, excluding others from social groups, or withdrawing friendships.^9^^,^^10^ One meta-analysis found a strong positive correlation (average *r* = 0.76) between relational and physical aggression.^11^ However, they have different gender patterns and psychosocial correlates; relational aggression is more prevalent among girls than boys,^12^ whereas physical aggression is more prevalent among boys than girls. Also, relational but not physical aggression is associated with internalizing psychopathology.^11^

Slow dissipation of aggression refers to the duration of children's anger or aggressive behavior. Slow dissipation of aggression is a relatively understudied but significant dimension of aggression that is associated with externalizing psychopathology. Specifically, children who exhibit slow dissipation of aggression tend to have peer problems, hold grudges, stay angry longer, and try to get back at others.^13^, ^14^, ^15^ Slow dissipation of aggression seems to be especially problematic among children with comorbid ADHD and oppositional defiant disorder or conduct disorder.^14^^,^^16^

Despite considerable research on 1 or 2 aspects of aggression, studies examining multiple aspects simultaneously and comprehensively remain scarce.^17^ Furthermore, research using mother reports to study aggression is remarkably limited despite the nearly universal use of mother ratings in clinical practice, with most studies relying on child self-report^18^ or teacher report.^19^ Meta-analyses highlight this underuse, revealing that only 2.8% and 7.6% of studies used mother reports for measuring reactive/proactive and relational aggression, respectively.^6^^,^^11^ This underuse is particularly surprising considering mothers’ prominent role in children’s development and their involvement in seeking services. Addressing this gap is crucial to gaining a more comprehensive understanding of aggression in children.

Another critical objective is to examine the associations between these various aspects of aggression and other aspects of youth conduct problems. Just as dissecting the heterogeneity within aggression is crucial, so is examining heterogeneity within broader conduct problems. Affective components, such as irritability (IRR) and callous-unemotional traits (CU) have emerged as significant factors in studying conduct problems in youth. Children with IRR are prone to anger and overreactive to perceived slights and disagreements.^20^ In addition, individuals with high IRR often exhibit deficits in emotional processing and instrumental learning.^21^^,^^22^ Studies consistently show significant associations between IRR and numerous adverse outcomes, including internalizing psychopathology, impairment, and peer problems.^23^ CU, characterized by a lack of guilt, low empathy, and shallow affect,^24^ are significantly associated with externalizing psychopathology, impairment, and peer problems.^25^ Furthermore, research suggests that the conduct problems in youth with CU may have distinct etiological profiles compared to those in other youth with conduct problems.^26^^,^^27^

Only 2 studies have comprehensively investigated all 4 aggression characteristics (reactive, proactive, relational, and slow dissipation) within the same sample. Waschbusch *et al.* (2004)^28^ used exploratory factor analysis to examine data from community-recruited children 5 to 12 years of age, including 1,549 children rated by teachers and 834 rated by parents. They found that peer-directed aggression and CU formed 1 factor, whereas adult-directed oppositional behavior (including irritability) and reactive aggression formed another. Both factors were significantly associated with ADHD, ODD, CD, and impairment. Building on these findings, Waschbusch *et al.* (2020)^29^ analyzed parent and teacher ratings of 219 clinic-recruited children 5 to 12 years of age. They reported that each aggression type (reactive, proactive, relational, slow dissipation) individually demonstrated significant associations with CU and IRR. When controlling for CU, ADHD, and defiance, however, IRR was uniquely associated with reactive aggression and slow dissipation. When controlling for IRR, ADHD, and defiance, CU were uniquely associated with proactive aggression and slow dissipation. No other studies have examined these diverse aggression types simultaneously.

The association between subtypes of aggression and affective components such as CU and IRR may vary based on the child’s sex. Notably, the concept of relational aggression emerged partly from the need to better understand aggression in girls.^10^ Crick and Grotpeter and Crick *et al.* proposed that boys and girls have similar levels of aggression, but that boys express it physically whereas girls express it socially or relationally.^9^^,^^30^ However, research on sex differences in aggression yields conflicting findings. A meta-analysis found significantly higher physical aggression in boys than in girls but no significant sex differences in relational aggression.^11^ Conversely, a contemporaneous narrative review concluded that relational aggression is more prevalent in girls.^12^ Interestingly, no studies have explored sex differences in the dissipation of aggression.

This study investigated the unique characteristics of reactive, proactive, relational, and slow dissipation of aggression in children 5 to 12 years of age using maternal ratings. It explored the relationships between these constructs, CU, IRR, demographic factors, psychopathology, and functional impairment. In addition, it examined the moderating role of sex in the associations among CU, IRR, and the 4 aggression types. This comprehensive examination of aggression and its related factors aims to inform the development of more effective interventions for children struggling with aggression.

## Method

### Sample and Procedure

A total of 508 children, ranging in age from 5 to 12 years (mean = 8.02, SD = 1.99), participated in this study. Participants were consecutive referrals to an outpatient mental health clinic focused on assessing and treating attention and behavior problems in youth. All children underwent a diagnostic evaluation by a licensed PhD psychologist. The assessment included a diagnostic interview with the parents,^31^ parent and teacher ratings, a review of educational and health records, administration of psychological tests (IQ,^32^ achievement,^33^ neuropsychological), and clinical observations of the child. Diagnoses were determined using all available information and applying criteria in the *DSM-5*.^34^ Table 1 summarizes the sociodemographic and diagnostic characteristics of the sample. The Penn State Institutional Review Board approved the use of these clinical assessment data for research purposes, with the requirement of informed consent/assent waived because of the retrospective analysis of existing clinical records. As part of the evaluation, ratings were collected from the child’s primary female caregiver, referred to as the mother in the remainder of this study.

**Table 1:** Descriptive Statistics for Sample

Variable	n	%
Sex at birth		
Female	183	36.0
Male	325	64.0
Race		
African American/Black	20	3.9
American Indian/Alaskan Native	1	0.2
Asian	5	1.0
Native Hawaiian/Other Pacific Islander	1	0.2
White	425	83.7
Other/Mixed	55	10.8
Unknown	1	0.2
Ethnicity		
Hispanic/Latino/Latina	51	10.0
Not Hispanic/Latino/Latina	457	90.0
Unknown		
Relationship to mother		
Biological	448	88.2
Adopted	29	5.7
Foster	2	0.4
Step	9	1.8
Other	20	3.9
Household income		
<$50,000	287	56.5
$50,000 to <$100,000	145	28.5
≥ $100,000	51	10.0
Unknown	25	4.9
Mother’s education		
Without HS diploma	13	2.6
HS graduate without a college education	73	14.4
Some college education	161	31.7
Degree from a 4-y college or more	257	50.6
Unknown	4	0.8
Psychiatric diagnoses		
ADHD	466	91.7
ODD	221	43.5
CD	71	14.0
Anxiety	55	10.8
Depression	4	0.8

Note: Psychiatry diagnoses were defined using criteria in the *DSM-5.*^34^ The diagnostic percentages do not sum to 100% because of comorbidity. ADHD = attention-deficit/hyperactivity disorder; CD = conduct disorder; HS = high school; ODD = oppositional defiant disorder.

### Measures

#### Nova Scotia Modified IOWA Conners

The Nova Scotia Modified IOWA Conners (NSIC) is a 34-item measure of externalizing behavior and peer functioning in youth.^28^ Each item is rated using a 4-point response scale ranging from 0 (not at all) to 3 (very much). The NSIC measures multiple constructs, including the 4 constructs of interest in this study: slow dissipation of aggression, relational aggression, proactive aggression, and reactive aggression. Each construct is measured using 3 items. The 3 items assessing reactive aggression (when teased, strikes back; blames others in fights; overreacts angrily to accidents) and the 3 items assessing proactive aggression (uses physical force to dominate; gets others to gang up on peers; threatens and bullies others) were directly derived from previous research studies.^5^^,^^35^ Similarly, the 3 items assessing relational aggression (gets others to reject a peer; gossips about other children; purposely excludes other kids) were derived from past research studies,^36^ with slight modifications to enhance conciseness. The 3 items assessing slow dissipation of aggression (tries to get back at other kids; holds a grudge for a long time; stays angry for a long time) were newly developed for the NSIC to measure the extent to which children persist in displaying anger and aggression.

#### Inventory of Callous–Unemotional Traits

The Inventory of Callous–Unemotional Traits (ICU) is a 24-item measure of callous-unemotional traits in youth.^37^ Each item is rated using a 4-point response scale that ranges from 0 (not at all) to 3 (definitely true). After reverse scoring as needed, items were summed to compute a CU score (mean = 23.94, SD = 11.73, α = 0.89).

#### Affective Reactivity Index

The Affective Reactivity Index (ARI) is a 7-item measure of irritability in youth.^38^ Each item is rated using a 3-point response scale that ranges from 0 (not true) to 2 (certainly true). Items were summed to compute an overall irritability score (mean = 4.39, SD = 3.53, α = 0.89).

#### Impairment Rating Scale

The Impairment Rating Scale (IRS) is a 10-item measure of children’s level of impairment across various domains, including peer relationships, sibling relationships, interactions with adults, academic performance, classroom behavior, self-esteem, functioning in the family, and overall adjustment.^39^ Respondents rated the questions on visual analog scales, with 1 end labeled as “no problem” and the other as “extreme problem.” An extra item assesses the child’s need for additional treatment or services, using a visual analog scale ranging from “no need for treatment or services” to “extreme need for treatment or services.” All items were scored on a metric ranging from 0 to 6. The items assessing problem areas were averaged to derive an overall impairment score (mean = 2.71, SD = 1.17, α = 0.80). The item assessing the need for treatment or services was examined separately (mean = 3.94, SD = 1.65).

#### Disruptive Behavior Disorders Rating Scale

The Disruptive Behavior Disorders Rating Scale (DBD) is a 45-item measure that assesses the *DSM-5* symptoms of ADHD, ODD, and CD.^40^^,^^41^ Respondents rate each item using a 4-point scale, ranging from 0 (not at all) to 3 (very much). Items were averaged within the following domains: ADHD-inattention (ADHD-inat; mean = 1.91, SD = 0.65, α = 0.86), ADHD-hyperactive/impulsive (ADHD-hypimp; mean = 1.72, SD = 0.69, α = 0.86), ODD (mean = 1.24, SD = 0.75, α = 0.89), and CD (mean = 0.22, SD = 0.26, α = 0.75).

### Statistical Analysis

First, an exploratory factor analysis (EFA) was computed to investigate the distinctiveness of reactive, proactive, relational, and slow dissipation of aggression. The EFA used the relevant items from the NSIC and was performed using MPLUS version 8.9.^42^ The weighted least squares mean and variance (WLSMV) estimation method was used, as it is appropriate for ordinal data such as the NSIC items. Based on the *a priori* model, a 4-factor solution was extracted from the EFA. Oblique (geomin) rotation was used to account for potential correlations among the factors. Model fit was evaluated using root mean square error of approximation (RMSEA), standardized root mean square residual (SRMR), and comparative fit index (CFI). Acceptable model fit was indicated by RMSEA and SRMR values ≤0.05 and CFI ≥0.95.^43^^,^^44^ Factor scores obtained from the EFA were extracted and used in subsequent regression analyses. The reliability of each factor score was estimated using omega and coefficient H.^45^ Second, individual differences in aggression types were examined by computing simple and partial correlations between aggression factor scores and demographic, psychopathology, and impairment scores, as well as CU and IRR scores. The partial correlations controlled for other types of aggression (eg, slow dissipation of aggression controlling for relational, proactive, and reactive aggression). Correlations were computed using SPSS version 28. Third, sex differences in aggression subtypes were investigated through ordinary least-squares regression analyses, with CU, IRR, sex (0 = girl, 1 = boy), and their interactions as independent variables and aggression scores as the dependent variables. These regression analyses were computed using the PROCESS macro version 4.3 in SPSS version 28.^46^ In cases of significant interactions, simple slope tests were conducted at the 16th and 84th percentiles of the sample.

## Results

### Exploratory Factor Analysis

The 4-factor EFA fit the data well; χ^2^(24) = 59.94; RMSEA = 0.054; CFA = 0.994; SRMR = 0.020. The standardized factor loading, presented in Table 2, indicated that the factors corresponded to slow dissipation, relational, proactive, and reactive aggression, and the reliability estimates of factor scores were acceptable. Factor scores were significantly correlated (*p* values <.001), ranging from 0.41 to 0.63. Specifically, the correlations between the following factors were identified: slow dissipation with relational, *r* = 0.48; slow dissipation with proactive, *r* = 0.41; slow dissipation with reactive, *r* = 0.58; relational with proactive, *r* = 0.44; relational with reactive, *r* = 0.55; and reactive with proactive, *r* = 0.63.

**Table 2:** Factor Loadings (Geomin Rotated) From the Exploratory Factor Analysis of the Nova Scotia IOWA Conners Items

Item	Slow dissipation	Relational	Proactive	Reactive
Tries to get back at other kids	0.203∗	0.200∗	0.276∗	**0.407**∗
Holds a grudge for a long time	**0.729**∗	0.153∗	–0.051	0.161
Stays angry for a long time	**0.964**∗	–0.007	0.084	–0.012
Gets others to reject a peer	0.136	**0.847**∗	–0.011	–0.018
Gossips about other children	0.007	**0.823**∗	–0.160	0.125
Purposely excludes other kids	–0.111	**0.769**∗	0.148	0.122
Uses physical force to dominate	0.023	–0.020	**0.773**∗	0.199
Gets others to gang up on peers	0.039	**0.817**∗	0.281	–0.127
Threatens and bullies others	–0.013	0.290∗	**0.675**∗	0.049
When teased, strikes back	0.018	0.103	0.200	**0.586**∗
Blames others in fights	0.001	0.029	–0.015	**0.895**∗
Overreacts angrily to accidents	0.327∗	–0.105	0.050	**0.549**∗
Reliability estimates				
Coefficient H	0.935	0.892	0.722	0.841
Omega	0.872	0.940	0.800	0.872

Note: Boldface type indicates the highest factor loading for each item.

∗ Factor loading significant at *p* < .05.

### Correlations

Table 3 presents the simple and partial correlations among aggression factor scores and demographic variables, psychopathology, impairment, CU, and IRR. Only correlations with *p* < .001 were interpreted as significantly different from zero because of the number of correlations computed. Simple correlations showed that aggression scores were significantly associated with ADHD-hypimp, ODD, and CD but (generally) not ADHD-inat. Moreover, aggression scores were significantly correlated with impairment, need for treatment, CU, and IRR, aligning with the expected pattern. Demographic factors and IQ were not correlated with any aggression scores, except for a small correlation between younger age and higher proactive aggression. Among the 72 partial correlations examined, 9 were significant, indicating that these aspects of aggression were uniquely associated with outcomes after accounting for the other aspects of aggression.

**Table 3:** Simple and Partial Correlations Between Aggression Scores and Other Variables

	Simple correlations				Partial correlations			
	Slow dissipation	Relational	Proactive	Reactive	Slow dissipation	Relational	Proactive	Reactive
Age	–0.03	0.03	–0.17∗	–0.04	–0.03	0.13	–0.24∗	0.10
Male sex	0.10	–0.07	0.11	0.11	0.08	–0.24∗	0.07	0.10
Hispanic ethnicity	–0.01	0.01	0.00	–0.02	0.00	0.03	0.02	–0.04
Minority race	–0.07	–0.01	–0.02	–0.02	–0.08	0.03	0.00	0.02
IQ scores^32^								
Verbal	0.10	–0.01	0.00	0.05	0.09	–0.08	–0.06	0.05
Perceptual reasoning	–0.01	–0.08	–0.06	0.01	–0.02	–0.11	–0.10	0.14
Full scale	0.06	–0.03	–0.02	0.06	0.03	–0.11	–0.09	0.12
Achievement test scores^33^								
Reading	0.01	–0.09	–0.06	–0.02	0.04	–0.11	–0.06	0.06
Mathematics	0.07	–0.07	–0.02	0.04	0.08	–0.14	–0.07	0.10
Spelling	0.07	–0.06	–0.01	0.05	0.06	–0.14	–0.07	0.11
DBD Rating Scale scores^40^								
ADHD-hyper/impulsive	0.25∗	0.29∗	0.27∗	0.32∗	0.02	0.09	0.02	0.10
ADHD-inattention	0.14	0.14	0.07	0.17∗	0.01	0.04	–0.12	0.14
ODD	0.60∗	0.52∗	0.58∗	0.68∗	0.23∗	0.01	0.07	0.28∗
CD	0.45∗	0.51∗	0.63∗	0.57∗	0.05	0.12	0.32∗	0.04
Impairment Rating Scale^39^								
Average impairment	0.38∗	0.35∗	0.37∗	0.41∗	0.11	0.02	0.07	0.18∗
Need for treatment	0.27∗	0.24∗	0.30∗	0.32∗	0.07	0.01	0.08	0.07
Affect CP Scores								
Irritability^38^	0.68∗	0.46∗	0.54∗	0.63∗	0.43∗	–0.08	0.10	0.17∗
Callous-unemotional^37^	0.37∗	0.38∗	0.43∗	0.47∗	0.04	0.04	0.09	0.16∗

Notes: Partial correlations controlling for other types of aggression (eg, slow dissipation of aggression controlling for relational, proactive, and reactive aggression). CP = conduct problems.

∗ Significantly different from zero at *p* < .001.

### Regressions

#### Slow Dissipation of Aggression

**Figure 1:**
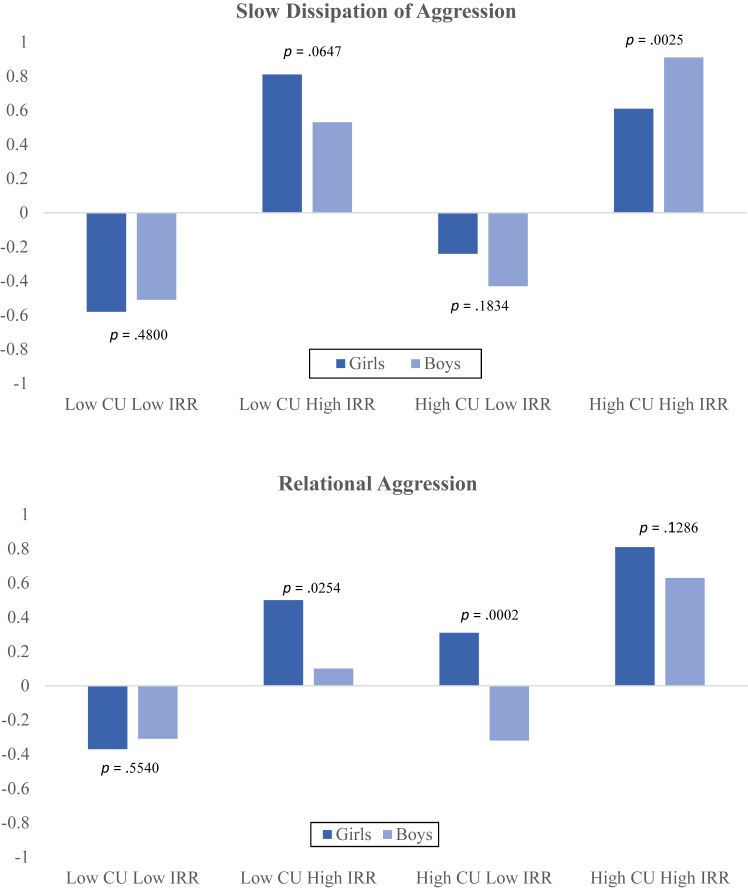
Graphical Representation of the CU∗Irritability∗Sex Interaction for Slow Dissipation of Aggression (Top) and Relational Aggression (Bottom) ***Note:****Low and high values of CU and irritability were defined using values at the 16th and 84th percentiles. CU = callous-unemotional traits; IRR = irritability.*

There was a significant 3-way interaction among CU, IRR, and sex (Table 4). A plot of the interaction and simple slope tests (Figure 1) indicated that boys displayed significantly slower dissipation of aggression than girls (*p* = .0025) when both IRR and CU were high.

**Table 4:** Regressions of Aggression Subtypes on Irritability, Callousness, Sex, and Their Interactions

	Aggression Subtypes			
	Slow Dissipation	Relational	Proactive	Reactive
Intercept	–0.750∗	–0.718∗	–0.853∗	–1.129∗
Callousness	0.014∗	0.029∗	0.020∗	0.027∗
Irritability	0.185∗	0.118∗	0.113∗	0.165∗
Sex (0 = girl, 1 = boy)	0.200	0.416∗	0.327	0.269
Irritabilty∗callousness	–0.003∗	–0.002	–0.001	–0.002
Callousness∗sex	–0.011	–0.029∗	–0.010	–0.012
Irritability∗sex	–0.086∗	–0.103∗	–0.052	–0.058
Callousness∗irritability∗sex	0.004∗	0.004∗	0.002	0.003
Overall model				
*F* (df = 7, 500)	67.45	29.92	38.74	60.37
*R*^2^	0.49	0.30	0.35	0.46
*p*	<0.0001	<0.0001	<0.0001	<0.0001

Notes: Values in the table are unstandardized regression coefficients.

∗ Significantly different from zero at *p* < .05.

#### Relational Aggression

There was a significant 3-way interaction among CU, IRR, and sex (Table 4). A plot of the interaction and simple slope tests (Figure 1) showed that girls displayed higher relational aggression when IRR was high and CU was low, as well as when CU was high and IRR was low. In contrast, both boys and girls had low relational aggression when both CU and IRR were low, and both sexes had high relational aggression when both CU and IRR were high.

#### Proactive Aggression

After controlling for other variables, higher CU and IRR scores were significantly associated with higher proactive aggression scores (Table 4).

#### Reactive Aggression

After controlling for other variables, higher CU and IRR scores were significantly associated with higher reactive aggression scores (Table 4).

## Discussion

Childhood aggression is a harmful behavior with significant consequences for both perpetrators and victims. Although various frameworks differentiate between types of aggression, such as reactive, proactive, relational, and slow dissipation of aggression, limited research has examined them collectively. Furthermore, scant knowledge exists regarding the association between these aspects of aggression, constructs such as IRR and CU, and potential differences between boys and girls. This study addressed these gaps in the literature.

The exploratory factor analysis supported the proposed distinctions among aspects of aggression. The data fit a 4-factor model representing reactive, proactive, relational, and slow dissipation of aggression. This result confirms that these aggression components are distinct but correlated, aligning with previous research.^29^ These results confirm the independence of these aggression components and highlight their interconnectedness. Notably, this study provides the first empirical evidence for these 4 aggression components when examined together, isolated from other externalizing behaviors. This novel approach paves the way for a more nuanced understanding of childhood aggression and its underlying mechanisms.

Although establishing the factorial validity of the aggression subtypes was crucial, exploring their associations with other constructs was equally important. Sex was associated with relational aggression, with girls exhibiting higher relational aggression scores than boys, but only in partial correlations, suggesting that sex differences in relational aggression become apparent only after accounting for other aggression types. This finding may partially explain relational aggression sex difference discrepancies in previous research,^10^ and provides a more nuanced understanding of sex differences in relational aggression. Age was associated with proactive aggression in both simple and partial correlations, with younger children displaying higher levels of proactive aggression. This pattern is consistent with longitudinal data.^47^ Furthermore, all 4 aggression types—reactive, proactive, relational, and slow dissipation—were associated with ADHD-hyperactivity/impulsivity, ODD, and CD measures, supporting the link between aggression and externalizing psychopathology.^48^ Interestingly, partial correlations revealed that slow dissipation and reactive aggression were uniquely correlated with ODD, suggesting that the intensity and duration of children’s reactions to provocation are crucial for understanding ODD. Notably, reactive aggression stood out as the only aspect of aggression uniquely associated with impairment, underscoring its crucial role in children’s overall adjustment. In addition, proactive aggression emerged as uniquely associated with CD, mirroring prior research findings.^7^^,^^49^^,^^50^ This emphasizes the importance of considering specific aggression types when evaluating and managing not only children with ADHD, ODD, and CD but also aggression in other children, such as children with autism spectrum disorders.

Simple correlations also revealed significant correlations between CU and IRR and each type of aggression. However, partial correlations revealed that IRR was uniquely associated with slow dissipation of aggression and reactive aggression. Notably, the partial correlation between the slow dissipation of aggression and IRR was nearly 3 times larger, highlighting the importance of considering this aggression component. Additionally, CU was uniquely associated with reactive aggression, aligning with previous findings.^7^^,^^16^^,^^29^ Surprisingly, we found no unique association between CU and proactive aggression, contrasting with previous studies.^16^^,^^51^ This suggests that controlling for other aggression subtypes may influence the observed association between CU and proactive aggression, a finding that suggests the need for further investigation.

Regression analyses further explored the individual and combined effects of CU, IRR, and sex on aggression subtypes. These results revealed significant associations between both CU and IRR and each type of aggression, even after controlling for each other and sex. Significant interactions between CU, IRR, and sex emerged for both slow dissipation and relational aggression. These interactions showed that boys exhibited greater difficulty letting go of anger when both CU and IRR were high, whereas girls exhibited elevated relational aggression when either CU or IRR was high. These findings underscore robust and independent contributions of CU and IRR to different aggression subtypes, further highlighting the importance of considering the interplay between these constructs and sex in understanding and predicting childhood aggression.

This study possesses several strengths, including a large sample size, inclusion of multiple types of aggression, incorporation of maternal ratings, and use of empirically validated measures. However, some limitations warrant consideration. The study’s cross-sectional design restricts the establishment of causal relationships between variables, necessitating future longitudinal studies. In addition, potential biases may arise from using a single informant (mother) for aggression assessment. The generalizability of the findings is limited to the studied population of children referred for mental health evaluation or treatment, potentially limiting their applicability to other age groups or cultural contexts. The sample arguably represents youth seeking clinical services rather than the broader community. Future research should investigate the generalizability of these results to community samples and explore how sociodemographic factors beyond sex influence aggression subtypes. Furthermore, although this study examined CU and irritability as predictors of aggression subtypes, it did not incorporate other potentially relevant factors, such as family dynamics or genetic influences. Moreover, the sample’s male-to-female ratio was approximately two-thirds to one-third. Although this distribution aligns with typical referral patterns for attention and behavior problem assessments,^52^ its potential impact on the results remains unclear. Finally, potential measurement overlaps between items measuring aggression subtypes and constructs such as CD or ODD may introduce confounds, requiring careful interpretation of the results.

This study sheds valuable light on the distinct subtypes of aggression in children and their links to CU and IRR, illuminating the multifaceted nature of childhood aggression. These findings hold relevance for child and adolescent psychiatry, as they underscore the need to consider multiple aggression dimensions, including reactive, proactive, relational, and slow dissipation, when assessing and treating youth exhibiting aggressive behaviors. For example, the observed sex/gender differences highlight the importance of tailoring prevention and intervention strategies to specific subgroups, such as addressing IRR or CU to reduce relational aggression in girls. These results also emphasize the importance of personalized treatment approaches, building upon research demonstrating promising outcomes of customized interventions based on individual CU and IRR levels.^53^, ^54^, ^55^, ^56^ Integrating these findings into current clinical practice holds significant promise. By incorporating different aggression dimensions into assessments and using the results to tailor treatment plans, clinicians can enhance case conceptualization and therapeutic efficacy, ultimately leading to improved outcomes for youth struggling with aggressive behaviors.

Future research should explore the longitudinal trajectories of aggression subtypes, explore potential mediating and moderating factors, consider contextual factors (eg, home vs school setting), and investigate the interplay between genetic and environmental influences. In addition, incorporating diverse populations and cultural contexts into future research will broaden the generalizability of findings and facilitate the development of culturally sensitive approaches to prevention, assessment, and treatment. Addressing these research gaps can advance our understanding of childhood aggression and pave the way for more effective strategies that promote children’s well-being and optimal functioning.
